# Aboriginal premature mortality within South Australia 1999-2006: a cross-sectional analysis of small area results

**DOI:** 10.1186/1471-2458-11-286

**Published:** 2011-05-10

**Authors:** David Banham, Heather Jury, Tony Woollacott, Robyn McDermott, Fran Baum

**Affiliations:** 1 Research and Ethics Policy, SA Health, 11 Hindmarsh Square, Adelaide, SA, 5000, Australia; 2 SA NT DataLink and Division of Health Sciences, University of South Australia, GPO Box 2471, Adelaide, South Australia 5001, Australia; 3 Southgate Institute, Faculty of Health Sciences, Flinders University, GPO Box 2100, Adelaide, South Australia 5001, Australia

## Abstract

**Background:**

This paper initially describes premature mortality by Aboriginality in South Australia during 1999 to 2006. It then examines how these outcomes vary across area level socio-economic disadvantage and geographic remoteness.

**Methods:**

The retrospective, cross-sectional analysis uses estimated resident population by sex, age and small areas based on the 2006 Census, and Unit Record mortality data. Premature mortality outcomes are measured using years of life lost (YLL). Subsequent intrastate comparisons are based on indirect sex and age adjusted YLL results. A multivariate model uses area level socio-economic disadvantage rank, geographic remoteness, and an interaction between the two variables to predict premature mortality outcomes.

**Results:**

Aboriginal people experienced 1.1% of total deaths but 2.2% of YLL and Aboriginal premature mortality rates were 2.65 times greater than the South Australian average. Premature mortality for Aboriginal and non-Aboriginal people increased significantly as area disadvantage increased. Among Aboriginal people though, a significant main effect for area remoteness was also observed, together with an interaction between disadvantage and remoteness. The synergistic effect shows the social gradient between area disadvantage and premature mortality increased as remoteness increased.

**Conclusions:**

While confirming the gap in premature mortality rates between Aboriginal South Australians and the rest of the community, the study also found a heterogeneity of outcomes within the Aboriginal community underlie this difference. The results support the existence of relationship between area level socio-economic deprivation, remoteness and premature mortality in the midst of an affluent society. The study concludes that vertically equitable resourcing according to population need is an important response to the stark mortality gap and its exacerbation by area socio-economic position and remoteness.

## Background

Indigenous people around the world experience poorer mortality outcomes than their non-Indigenous contemporaries [1]. The average life expectancy of Aboriginal Australians at birth is almost 11 years less than that of the general population [2] and this difference is larger than others in similarly developed settings [1]. In response, the Council of Australian Governments (COAG) have formally committed to 'Closing the gap', a strategy to reduce areas of Aboriginal disadvantage [3] including life expectancy.

Aboriginal mortality outcomes are not homogenous though and regularly reported data shows variations in outcomes between jurisdictions. For example, in South Australia the difference between Aboriginal and non-Aboriginal life expectancy is about 13 years [2,4]. Less frequent reporting also describes important variations by area remoteness [5] and within jurisdictions [6,7]. Of course, within group variations in population health outcomes disaggregated by socio-economic status are also frequently reported [8,9]. However, a recent examination of Australian population health data showed how the use of a single area measure fails to adequately inform planning on issues such as health workforce training and incentives [10]. A more fruitful approach is to combine analysis of area socio-economic disadvantage and remoteness [9,11].

There is an increasing need to inform: health priority setting [12]; appropriate resourcing [10]; and, tailored preventive and service responses that are relevant to population need, by providing comprehensive information about small area variations in health status. The South Australian Aboriginal Health Care Plan [13] responded to such need by profiling each of 11 smaller, intrastate regions on a range of health related indicators.

The Plan used premature mortality to assess the mortality burden using Years of Life Lost (YLL) whereby all deaths contribute YLL, but the younger the age at death, the greater the YLL number. A separate brief report outlines results by region and details the amount and leading causes of premature mortality for South Australia and each of the Aboriginal Health Care Plan regions [14]. These regional results illustrate the considerable diversity in the health and wellbeing of Aboriginal people. For example, within the Adelaide metropolitan area loss from premature mortality in the western suburbs is almost double that of southern suburbs. In other parts of the state, regional outcomes are even more disparate ranging from better outcomes among Aboriginal people in the South-East and Peri-Urban ring surrounding Adelaide to areas such as the West Coast with four-fold higher rates of loss.

This paper aggregates mortality data for the period 1999 to 2006 to enable description of premature mortality outcomes at the smallest geographic level currently possible. After initially describing premature mortality outcomes for Aboriginal and non-Aboriginal South Australians, the analysis then examines how these outcomes vary for each sub-population across area level socio-economic disadvantage and geographic remoteness.

## Methods

Estimated resident population (ERP) for Aboriginal South Australians in the 2006 Census year [15] came from the Australian Bureau of Statistics (ABS) as did the most closely aligning 'low series' population estimates for the preceding years, 1999-2005 [16]. The 2006 estimates also included age and sex profiles by rurality, and total population for Statistical Local Areas (SLAs), the relatively small geographic areas used in intrastate analysis [17]. These profiles formed the basis for interpolating 1999-2005 Aboriginal ERP by age, sex and SLA. The difference between Aboriginal population estimates and the yearly total ERP from annual ABS releases [18] were used for enumerating non-Aboriginal people. The mean annual total population for each SLA was 12 216 (SD = 9 672) and ranged from 0 to 34 797.

Outcomes data were drawn from unit level mortality records processed within South Australia's summary population health measurement series for the years 1999 to 2006 [19]. Premature mortality for each death is expressed using years of life lost (YLL), which is the remaining expected, standard life expectancy [20] at age of death, time discounted at an annual rate of 3 percent. For example, if a death occurs at age 50 and the average life expectancy at 50 is 34 years, then this death contributes 34 YLL, or approximately 20 YLL after time discounting.

Deaths where Aboriginality, defined as Aboriginal and/or Torres Strait Islander, was not stated or unknown were categorised as non-Aboriginal. The deceased's place of usual residence is a mandatory field using SLAs. Changes in statistical geography during the observation period saw several new SLAs created. For example, Anangu Pitjantjatjara and Maralinga Tjarutja SLAs were partitioned from the rest of Unincorporated Far North SLA in 2005. Deaths occurring in those areas from 1999 to 2004 show Unincorporated Far North as place of usual residence, so for the purpose of this analysis the three SLAs were collapsed into one.

The ABS rank Australia's populated SLAs by their socio-economic characteristics [21]. Of the four composite index scores available for statistical tests, and despite including Aboriginality in its construction, the Index of Relative Socio-economic Disadvantage (IRSD) [21] is most frequently used in South Australian analyses [8,9] because of its predictive validity. SLAs also have a measure of geographic remoteness, ARIA+, ranging from 0 (high accessibility) to 15 (high remoteness) as determined by road distance to service centres [17,22]. Additional file 1 Appendix 1 details SLA remoteness level and disadvantage ranking. SLAs with nominal population, no recorded deaths and no relative IRSD rank would not contribute to the analysis and were omitted.

As SLA age and sex profiles vary and Aboriginal case and population numbers are often small, population and YLL figures were collapsed into age categories of 0-4 then 10 year age groups to 55+ [23] before indirectly age and sex adjusting results [24] using Stata version 11.1 [25] to enable comparison across areas. SLA outcomes represent the ratio of observed versus expected premature mortality as based on the South Australian total for 1999 to 2006. For example, an outcome of 150 indicates the SLA's ratio of observed versus expected premature mortality was one and a half times, or 50% higher, than the South Australian average after adjusting for sex and age differences.

Cross tabulations describe population and YLL distribution across demographic variables, then standardised YLL ratios by area level disadvantage and remoteness. Relationships between area level outcomes of IRSD ranking (where 1 = least disadvantage upwards) and remoteness categories [11] (where 0 = Major cities with ARIA+ < = 0.2; 1 = Regional, ARIA+ > 0.2 & < = 5.92; 2 = Remote, ARIA+ > 5.92) and the possible interaction between area disadvantage and remoteness were examined graphically. Models were then derived for Aboriginal and non-Aboriginal populations using least squares regression with each SLA's contribution weighted according to their population size. The final model was selected on the basis of the best fit to Aboriginal outcomes and ease of explanation. Outlying SLAs were tested for removal at p = 0.05 [26] if detected. Given the relatively small numbers underlying the analysis and a small positive skew in residuals, standard errors robust to heteroskedasticity were used [27]. Variance inflation factors were also examined using an upper value of 10 to indicate multicollinearity among predictor variables and undue influence on the least square estimates [28].

## Results

### Population

Aboriginal people comprise 1.7% of South Australia's population (Table 1) and their demographic profile varies markedly from the rest of the population in several ways. The Aboriginal community's age profile is much younger. Almost twice the proportion of Aboriginal people are aged under 15 years (36.6% compared to 18.7%) while less than 8% are aged 55 years or more (compared to over 25% in the rest of the community). When the total population is divided into disadvantage quintiles, half of Aboriginal South Australians live in the most disadvantaged areas and three-quarters in the two most disadvantaged quintiles. Additionally, Aboriginal people are also more likely to live in Regional areas than non-Aboriginal people (33.2% compared to 25.0%) and far more likely to live in Remote areas (18.8% and 3.5% respectively).

**Table 1:** Mean annual population distribution by area disadvantage, remoteness and Aboriginality in South Australia 1999-2006

			Area Remoteness (ARIA+)							
Aboriginal			Major cities		Regional		Remote		Total	
			N	Percent	N	Percent	N	Percent	N	Percent
	Age									
		0-4	1555	12.2%	1073	12.2%	516	10.4%	3144	11.9%
		5-14	3240	25.5%	2164	24.6%	1139	22.9%	6543	24.7%
		15-24	2674	21.0%	1697	19.3%	973	19.6%	5344	20.2%
		25-34	1763	13.9%	1204	13.7%	778	15.6%	3745	14.1%
		35-44	1553	12.2%	1104	12.6%	706	14.2%	3363	12.7%
		45-54	1072	8.4%	770	8.8%	467	9.4%	2309	8.7%
		55+	872	6.9%	776	8.8%	396	8.0%	2042	7.7%
	
	Area Disadvantage (2006 IRSD)									
		Q5 Least Disadvantage	1077	4.1%	181	0.7%	69	0.3%	1328	5.0%
		Q4	1612	6.1%	467	1.8%	123	0.5%	2202	8.3%
		Q3	2159	8.1%	945	3.6%	146	0.6%	3250	12.3%
		Q2	2988	11.3%	2136	8.1%	1169	4.4%	6292	23.8%
		Q1 Most Disadvantage	4893	18.5%	5058	19.1%	3467	13.1%	13418	50.7%
	
		Total	12728	48.0%	8788	33.2%	4974	18.8%	26490	100.0%

Non-Aboriginal										
			Major cities		Regional		Remote		Total	
	
	Age									
		0-4	60971	5.7%	23292	6.2%	3601	6.8%	87864	5.9%
		5-14	130165	12.1%	54464	14.5%	7721	14.6%	192350	12.8%
		15-24	150100	14.0%	43148	11.5%	5781	10.9%	199030	13.3%
		25-34	150983	14.1%	43859	11.7%	7170	13.5%	202012	13.5%
		35-44	158910	14.8%	57251	15.2%	8196	15.5%	224358	15.0%
		45-54	149555	14.0%	54636	14.5%	7610	14.3%	211801	14.1%
		55+	271048	25.3%	99060	26.4%	12964	24.4%	383072	25.5%
	
	Area Disadvantage (2006 IRSD)									
		Q5 Least Disadvantage	265311	17.7%	39752	2.6%	3936	0.3%	308999	20.6%
		Q4	227407	15.2%	57809	3.9%	7304	0.5%	292519	19.5%
		Q3	204166	13.6%	83724	5.6%	15335	1.0%	303225	20.2%
		Q2	185390	12.4%	104032	6.9%	20317	1.4%	309739	20.6%
		Q1 Most Disadvantage	189458	12.6%	90394	6.0%	6152	0.4%	286004	19.1%
	
		Total	1071732	71.4%	375711	25.0%	53044	3.5%	1500487	100.0%

### Deaths

Of 94 785 deaths among people who usually resided in South Australia in the years 1999 to 2006, 1041 (1.1%) were identified as Aboriginal. All but 176 of total death records referenced a known SLA within SA (117 people were living overseas at the time of death; 42 were of no fixed place; and 17 did not reside in a defined SLA). While Aboriginal people were over represented among these with 13 deaths (7.4%), the records were omitted from subsequent analyses because of the focus on results for small areas.

### Premature mortality

The 94 609 deaths featuring in this analysis were responsible for a total of 878 251 YLLs. Aboriginal deaths accounted for 19 665 (2.2%) of the total YLL accrued (Table 2).

**Table 2:** Association of death and premature mortality outcomes with demographic variables and Aboriginality in South Australia 1999-2006

		Aboriginality					
		Aboriginal		non-Aboriginal		Total	
		N	Percent	N	Percent	N	Percent
Deaths	Total	1028	1.1%	93581	98.9%	94609	100.0%

YLL	Total	19665	2.2%	858586	97.8%	878251	100.0%

Gender	Male	11316	54.7%	470460	49.4%	481776	49.5%
	Female	8349	40.3%	388126	40.8%	396475	40.8%

Age	0-4	1609	8.2%	20284	2.4%	21893	2.5%
	5-14	351	1.8%	4728	0.6%	5079	0.6%
	15-24	1710	8.7%	23634	2.8%	25344	2.9%
	25-34	2690	13.7%	34054	4.0%	36745	4.2%
	35-44	3921	19.9%	51884	6.0%	55805	6.4%
	45-54	4179	21.2%	86244	10.0%	90422	10.3%
	55+	5205	26.5%	637757	74.3%	642963	73.2%

Area Disadvantage	Q5 Least Disadvantage	408	2.1%	144135	16.8%	144544	16.5%
(2006 IRSD)	Q4	1034	5.3%	148964	17.3%	149997	17.1%
Quintile	Q3	1404	7.1%	176592	20.6%	177996	20.3%
	Q2	6836	34.8%	200686	23.4%	207522	23.6%
	Q1 Most Disadvantage	9984	50.8%	188209	21.9%	198193	22.6%

Area Remoteness	Major cities	7334	37.3%	610100	71.1%	617434	70.3%
(ARIA+)	Regional	5855	29.8%	219115	25.5%	224970	25.6%
	Remote	6476	32.9%	29371	3.4%	35847	4.1%

While the sex distribution of YLL by Aboriginality did not notably differ, the age distribution of YLL did vary markedly. The proportions of YLL from deaths at ages 0 to 54 years are up to 3 times higher among Aboriginal compared to non-Aboriginal people. The upper age category of 55 and over accounted for almost three quarters of non-Aboriginal YLL compared to around one-quarter for Aboriginal people.

YLL distribution also varied widely across socioeconomic disadvantage quintiles whereby YLL numbers increased with disadvantage, particularly among Aboriginal people. Aboriginal YLL was distributed fairly evenly across remoteness level but was highly concentrated in Major cities among non-Aboriginals.

### Standardised premature mortality ratios

Significant variations in standardised YLL ratios (SYLLR) exist between Aboriginal/non-Aboriginal groups and within Aboriginal results. Overall, Aboriginal YLL outcomes were 2.65 times that of the South Australian average (Table 3).

**Table 3:** Association of standardised YLL ratio (SYLLR) and area attributes by Aboriginality in South Australia 1999-2006

	Area Remoteness (ARIA+)											
Aboriginal	Major cities			Regional			Remote			Total		
Area Disadvantage (2006 IRSD)		95% Confidence intervals			95% Confidence intervals			95% Confidence intervals			95% Confidence intervals	
	SYLLR	Lower	Upper	SYLLR	Lower	Upper	SYLLR	Lower	Upper	SYLLR	Lower	Upper
Q5 Least Disadvantage	134	15	253	61	0	238	0	n.a	n.a	117	22	212
Q4	200	141	258	100	47	153	155	72	238	176	134	242
Q3	185	143	227	111	52	171	124	0	284	161	126	219
Q2	201	155	247	225	159	291	418	283	552	290	231	259
Q1 Most Disadvantage	270	195	346	257	214	300	580	445	716	305	245	330

Total	219	186	252	221	191	251	446	370	521	262	235	247

												
Non-Aboriginal	Major cities			Regional			Remote			Total		
		95% Confidence intervals			95% Confidence intervals			95% Confidence intervals			95% Confidence intervals	
	SYLLR	Lower	Upper	SYLLR	Lower	Upper	SYLLR	Lower	Upper	SYLLR	Lower	Upper

Q5 Least Disadvantage	85	71	100	66	56	75	70	n.a	n.a	83	71	94
Q4	89	79	99	87	76	99	90	76	99	89	82	95
Q3	95	87	102	95	90	95	102	86	104	95	91	99
Q2	110	97	110	103	97	109	96	88	118	106	100	112
Q1 Most Disadvantage	118	107	129	113	108	118	97	96	130	116	110	122

Total	98	92	104	97	92	102	95	89	102	98	94	102

Outcomes for Aboriginal people within areas of least disadvantage remained higher than the SA average. Premature mortality loss then increased as disadvantage increased with rates observed in areas of most disadvantage being three times those of South Australia overall. To a lesser extent, relative YLL rates increased as areas became more remote and this is particularly so when contrasting the two-fold difference between Remote and the other two categories.

The results also indicate an interaction between level of remoteness and socio-economic disadvantage because the range of Aboriginal SYLLRs increased markedly as remoteness category increased. For example, within Major Cities, ratios ranged from 133 for least disadvantage up to 271 for most disadvantage, a difference of 138. In Remote areas, no Aboriginal deaths were recorded in the least disadvantaged SLA while the most disadvantaged areas had a ratio of 580.

Non-Aboriginal outcomes also show a persistent, albeit smaller increase in premature mortality as disadvantage increased and nominal differences between remoteness levels.

The final multivariate models (Table 4) were significant for Aboriginal, F(3, 117) = 26.7, p < 0.001, and non-Aboriginal people alike, F(3, 117) = 15.14, p < 0.001. Using the predictor variables of area disadvantage ranking, remoteness category and an interaction term of remoteness category (squared) by disadvantage rank, the models accounted for 53.0% of the variance in Aboriginal SYLLRs and 34.6% of variance in non-Aboriginal outcomes.

**Table 4:** Modelled relationship of SLA attributes and standardised premature mortality rates by Aboriginality in South Australia 1999-2006

	Aboriginal				non-Aboriginal				
		95% Confidence Intervals				95% Confidence Intervals			
	β	Lower	Upper	p value	β	Lower	Upper	p value	Variance Inflation Factor
Constant	122.46	61.06	183.85	< 0.001	79.05	69.37	88.73	< 0.001	
Area Disadvantage (2006 IRSD) rank	1.27	0.41	2.13	< 0.01	0.35	0.23	0.47	< 0.001	1.230
(1 = least disadvantage; 121 = most disadvantage)									
Area Remoteness	-109.15	-171.70	-46.60	< 0.001	-3.97	-12.78	4.85	0.37	5.64
(0 = Major cities; 1 = Regional; 2 = Remote)									
Remoteness by Remoteness by Disadvantage	0.99	0.64	1.35	< 0.001	-0.01	-0.08	0.06	0.82	6.08

**Figure 1:**
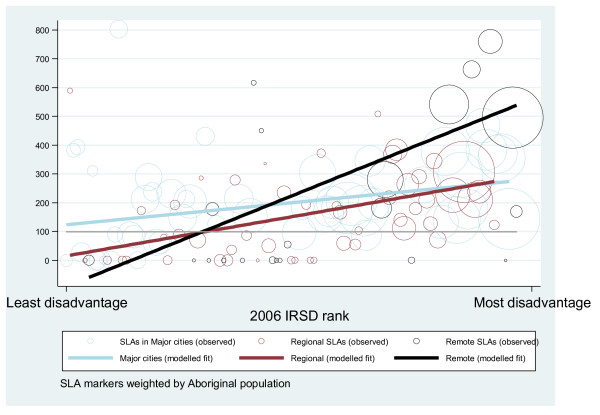
**Observed and modelled Aboriginal premature mortality outcomes by SLA in South Australia 1999-2006**.

**Figure 2:**
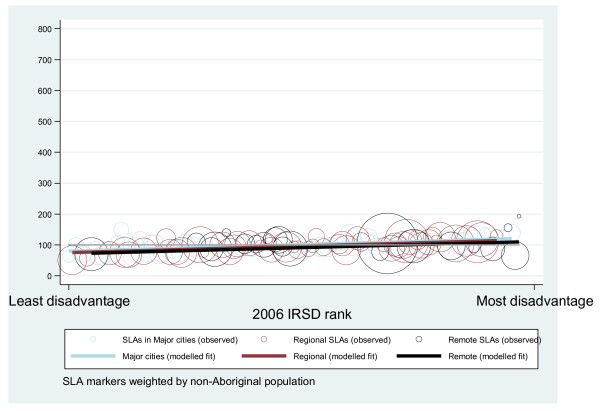
**Observed and modelled non-Aboriginal premature mortality outcomes by SLA in South Australia 1999-2006**.

The models indicate that increased disadvantage rank was associated with increased loss from premature mortality, regardless of Aboriginality. Among Aboriginal people though, a significant main effect for area remoteness was also observed. Aboriginal results also show a significant interaction between area disadvantage and remoteness. This interaction is synergistic, or reinforcing, and indicates that as area remoteness changed so too did the effect of increased disadvantage with a particularly strong effect in Remote areas. Figures 1 and 2 illustrate the results for Aboriginal and non-Aboriginal populations respectively. The figures display each SLA's outcome, disadvantage rank and remoteness category weighted by the relevant Aboriginal/non-Aboriginal population, then overlay the predicted results for each combination of disadvantage, remoteness and remoteness category (squared) by disadvantage rank.

## Discussion

The years of life lost because of premature mortality among Aboriginal South Australians occurred at more than two and a half times the rate of non-Aboriginal people after allowing for sex and age differences between the populations. This is consistent with national data [5] and clearly illustrates the magnitude of the Gap in mortality outcomes within South Australia.

The analysis also clearly demonstrates the heterogeneity of results for Aboriginal people in South Australia. The results highlight a range of premature mortality outcomes existing among Aboriginal South Australians and underlying the gap in outcomes between Aboriginal and non-Aboriginals. Nevertheless, while significant differences in outcomes among Aboriginal South Australians exist, wherever Aboriginal deaths occurred they were generally associated with higher rates of loss compared to South Australia as a whole.

The findings are consistent with a recent national study [5] of Aboriginal premature mortality in showing incremental increases in mortality related loss as remoteness (measured by the road distance to service centres) increased. However, South Australian Aboriginals experienced greater per capita loss in remote areas. While equivalent proportions of premature mortality were observed in remote areas (35% nationally and 33% in South Australia), a smaller proportion of Aboriginal South Australians live in remote areas (19% in South Australia and 25% in Australia).

The South Australian results also show a clear association between increased socio-economic disadvantage at an area level and increasing premature mortality. The gradient observed is consistent with national death rates among Aboriginals younger than age 65 [9]. For example, the ratio of loss in high to low disadvantage quintiles was 2.6 in South Australia and 2.3 in Australia.

The current results also indicate the relationship between premature mortality, area level disadvantage and remoteness category is not just additive. A synergy between the two ecological variables of disadvantage and remoteness was found whereby the socio-economic gradient of premature mortality loss became steeper as remoteness increased. Road traffic accidents, the third largest contributor to premature mortality among Aboriginal people in South Australia [14], illustrate how these factors can adversely effect mortality outcomes. Greater socio-economic disadvantage reduces the likelihood of safe, reliable transport and increases the risk of accident. Increased remoteness may be associated with poorer road conditions while adversely affecting access to emergency medical services in the event of an accident. Any, or all, of these factors can exacerbate mortality from road traffic accidents which contributes proportionately more loss in areas of greater disadvantage and/or remoteness [14].

The results suggest comparatively better outcomes for Aboriginal people in less disadvantaged and Regional areas. This is congruent with the Aboriginal Health Care Planning Regional profiles for Peri-Urban Adelaide and the South-East [14] where relatively lower rates of premature mortality were observed. However, such outcomes are experienced by less than 10% of Aboriginal people living in Regional/Remote areas of average or less than average disadvantage. Fully half of the Aboriginal population live in the most disadvantaged of areas; and, a quarter of these in the Remote areas of the state.

The findings have important implications. Wide ranging, complex mortality gaps raise questions about how best to respond: including, questions of how to equitably distribute resources according to need. Some health economists argue for vertical equity [29]. In this particular case, this means a response that is suitably weighted to overcome the influence of factors such as disadvantage and remoteness. Indeed, vertical equity is often advocated for use within government policy as a means of promoting equitable resource allocation [30] but has less history within health per se [31]. If it is preferable to implement a response of that kind, then what weights should apply? Recent reports demonstrate that per capita health expenditure for Aboriginal people is positively weighted and increases in remote, rural areas [32]. However, other analyses indicate a single factor model is limited. For example, in the case of adequately matching primary health care related workforce and training supply with population health need, it is more appropriate to include both remoteness and disadvantage [10]. Our findings suggest a vertically equitable response would take account of area level remoteness, socio-economic disadvantage and the synergy between these two factors. In turn the findings could help inform discussion about appropriate resourcing levels and distribution necessary to make outcomes more equitable and contribute to closing the gap in outcomes.

Significant amounts of premature mortality loss among Aboriginal South Australians can be categorised as potentially avoidable (70% compared to 41% among non-Aboriginal people) [14]. Many of the conditions are amenable to health interventions (such as prevention of chronic disease, suicide and trauma related injury), while other opportunities for preventing early loss of life lie in other sectors (such as housing improvement, road traffic mortality and education). This highlights the importance of South Australian Government initiatives like Health in All Policies [33] in encouraging all agencies to work to alleviate the social determinants of health disadvantage.

This current analysis adds to our understanding of the nature and distribution of premature mortality outcomes within the Aboriginal community in South Australia. However, several factors suggest caution in interpreting the findings. Firstly, it is important to guard against the ecological fallacy whereby area characteristics are attributed to individuals. For example, it does not necessarily follow that a person residing in a disadvantaged area is a disadvantaged individual. Also, Aboriginal data is statistically volatile because of small population numbers and death counts. As mentioned earlier, Aboriginal population estimates are 'experimental' in nature [15]. Revised enumeration from Census 2011 can potentially improve certainty of these estimates within each Australian jurisdiction. Under-identification of Aboriginal people in service and death records is also a longstanding difficulty and leads to underestimating premature mortality in the Aboriginal community. A recent ABS data linkage study applied new statistical techniques and indicated about 86% of South Australian Aboriginal deaths are correctly identified (85% nationally) [34]. Routinely applying these methods to South Australia's death records will eventuate in better identification and coverage. In turn, this will improve precision in describing the relationship between area characteristics and population outcomes.

Notwithstanding these issues, the models derived in this analysis used two available area level indicators and accounted for half the variance in Aboriginal outcomes. This highlights the need to improve our understanding and accounting of influences on Aboriginal health outcomes. Ideally this will incorporate information of greater relevance not just at an individual level, for example access to fresh water, but also at social and cultural levels, such as access to homeland living.

## Conclusions

This small area analysis of premature mortality outcomes draws attention to equity gaps requiring a policy response and "helps make the invisible visible" [35]. The results clearly describe the relationship between area level socio-economic deprivation, remoteness and early loss of life in the midst of an affluent society. Further enumeration making better use of linked administrative data can help us monitor the outcomes of initiatives to improve equity and reduce the Aboriginal mortality gap.

## Competing interests

The authors declare that they have no competing interests.

## Authors' contributions

DB maintains the South Australian burden of disease series, operationalised the study, performed data analysis, drafted and revised the manuscript. HJ contributed to data analysis and revising the manuscript. TW participated in the study design and manuscript revision. RM assisted with manuscript drafting and revision. FB conceived of the original research question and helped draft and revise the manuscript. All authors read and approved the final manuscript.

## Pre-publication history

The pre-publication history for this paper can be accessed here:

http://www.biomedcentral.com/1471-2458/11/286/prepub

## Supplementary Material

Additional file 1 **Aboriginal premature mortality outcomes and SLA attributes in South Australia 1999-2006**. Details each Statistical Local Area's: ARIA+ remoteness level; 2006 relative socioeconomic disadvantage ranking within South Australia; and standardised premature mortality ratio for the years 1999-2006. Click here for file
